# A Lignin Silver Nanoparticles/Polyvinyl Alcohol/Sodium Alginate Hybrid Hydrogel with Potent Mechanical Properties and Antibacterial Activity

**DOI:** 10.3390/gels10040240

**Published:** 2024-04-01

**Authors:** Jie Yu, Fangli Ran, Chenyu Li, Zhenxin Hao, Haodong He, Lin Dai, Jingfeng Wang, Wenjuan Yang

**Affiliations:** 1 Key Laboratory of Subsurface Hydrology and Ecological Effect in Arid Region of the Ministry of Education, School of Water and Environment, Chang’an University, Xi’an 710064, China; 17719682756@163.com; 2 Department of Environment and Health, Institute of Environmental and Operational Medicine, Tianjin 300050, China; 3 Tianjin Key Laboratory of Pulp and Paper, Tianjin University of Science and Technology, Tianjin 300457, China

**Keywords:** antibacterial hydrogel, Lig-Ag NPs, PVA and SA, *E. coli*, *S. aureus*

## Abstract

Antibacterial hydrogels have attracted significant attention due to their diverse applications, efficient antimicrobial properties, and adaptability to various environments and requirements. However, their relatively fragile structure, coupled with the potential for environmental toxicity when exposed to their surroundings for extended periods, may significantly limit their practical application potential. In this work, a composite hydrogel was synthesized with outstanding mechanical features and antibacterial capability. The hydrogel was developed through the combination of the eco-friendly and enduring antibacterial agent, lignin silver nanoparticles (Lig-Ag NPs), with polyvinyl alcohol (PVA) and sodium alginate (SA), in varying proportions. The successful synthesis of the hydrogel and the dispersed distribution of Lig-Ag NPs within the hydrogel were confirmed by various analytical techniques, including field emission scanning electron microscopy (SEM), energy-dispersive spectroscopy (EDS), mercury intrusion porosimetry (MIP), Fourier transform infrared spectroscopy (FT-IR), X-ray diffraction (XRD), and X-ray photoelectron spectroscopy (XPS). The formation of multiple hydrogen bonds between Lig-Ag NPs and the composites contributed to a more stable and dense network structure of the hydrogel, consequently enhancing its mechanical properties. Rheological tests revealed that the hydrogel exhibited an elastic response and demonstrated outstanding self-recovery properties. Significantly, the antibacterial hydrogel demonstrated effectiveness against *Escherichia coli* (*E. coli*) and *Staphylococcus aureus* (*S. aureus*), achieving a <5% survival of bacteria within 12 h. This study presented a green and straightforward synthetic strategy for the application of antibacterial composite hydrogels in various fields.

## 1. Introduction

The spread of pathogenic microorganisms in the environment and food has posed a significant threat to human health due to biological contamination [1,2,3]. Consequently, a variety of antibacterial materials have been developed to address this issue. Antibacterial hydrogels have garnered significant attention due to their exceptional biocompatibility and biodegradability among a diverse array of antibacterial materials [4,5]. The preparation of antibacterial hydrogels typically involves combining polymers and antibacterial materials to obtain a three-dimensional porous network structure with desired properties.

Polymer composites have the potential to address the limitations of individual materials and yield synergistic effects through deliberate preparation methods [6]. The use of polyvinyl alcohol (PVA), a synthetic polymer, and sodium alginate (SA), a natural polymer, has been extensively discussed in various fields such as wastewater treatment, drug delivery, tissue engineering, wound healing, and food packaging in recent years [7]. PVA exhibits outstanding characteristics, including non-toxicity, biocompatibility, high hydrophilicity, and the ability to form fibers and films, as well as chemical and mechanical resistance [8]. On the other hand, SA is a naturally occurring polysaccharide polymer that is extensively utilized because of its exceptional biocompatibility, rapid degradability, and cost-effectiveness [9]. Additionally, SA demonstrates inadequate mechanical properties and stability, which limits its potential applications [10]. Hybridizing PVA and SA has been widely discussed for the synthesis of materials with desirable properties. For example, Seok et al. prepared structurally stable 3D scaffolds by adding PVA to pure SA hydrogels, which can be used as candidates for implantable biodegradable scaffolds in the fields of tissue engineering and medicine [11]. Kamoun et al. and Zhang et al. presented PVA-SA hydrogel wound dressings by simple cross-linking physically, which were both tough and elastic, with sufficient biocompatibility and degradability [12,13].

As a promising antibacterial material, antimicrobial hydrogels can be categorized as inorganic nanoparticle-containing hydrogels, antibacterial agent-containing hydrogels, and hydrogels with intrinsic antibacterial capacity [14]. Inorganic nanoparticles effectively destroy bacteria by releasing heavy metal ions, damaging cell membranes, generating oxidative stress, etc. [15]. Silver nanoparticles (Ag NPs) have frequently been utilized for compounding with hydrogels in recent years [16,17,18,19]. Juby et al. synthesized a PVA/gum acacacia hydrogel loaded with Ag NPs by a one-pot method, and the inhibition zone experiments revealed that the hydrogel had a satisfactory antibacterial effect against gram-negative *Escherichia coli* (*E. coli*) [20]. Stojkovska et al. designed a SA microbead containing Ag NPs. Experimental evidence demonstrated that both dry and wet microspheres exhibited the ability to inhibit the growth of *Staphylococcus aureus* (*S. aureus*) and *E. coli.* Consequently, they have the potential to serve as soft tissue implants and antibacterial wound dressings in biomedical applications [21]. Furthermore, the combination of Ag NPs and PVA/SA matrix has the potential to enhance the stability of Ag NPs within the hydrogel. The immobilization of Ag NPs in the hydrogel was enhanced by the formation of a substantial quantity of hydrogen bonds between the two polymers. This increase in hydrogen bonding effectively restricted the uncontrollable diffusion of silver from the matrix [22]. Unfortunately, Ag NPs are known to elicit a broad range of cytotoxic effects in humans [23,24] and can lead to numerous ecological issues upon exposure of the silver nanocores to the environment [25,26]. Therefore, there is a need to develop more non-toxic and environmentally friendly strategies for antibacterial hydrogel synthesis.

Lignin silver nanoparticles (Lig-Ag NPs) are nanomaterials characterized by a core–shell structure comprising an Ag core and a lignin shell. Hydroxyl or methoxyl groups of phenol on lignin reduce Ag^+^ to Ag NPs, and then transform them into dynamically stabilized semiquinone radicals. The “green” lignin shell makes Lig-Ag NPs environmentally friendly [27] and biocompatible [28]. Simultaneously, due to the inherent antibacterial properties of lignin, it has been observed that Lig-Ag NPs have a comparable detrimental impact on bacterial viability as Ag NPs. In comparison to Ag NPs, Lig-Ag NPs exhibited superior dispersion and prolonged antibacterial properties attributed to their core–shell structure [29]. Furthermore, Han et al. prepared a piezoresistive sensor hydrogel by compounding PVA and Lig-Ag NPs which has excellent elasticity and toughness attributed to the nanophase separation and strong molecular sacrificial bonding between PVA and Lig-Ag NPs in the internal structure of the hydrogel [30].

This study presents a straightforward approach for the synthesis of a composite hydrogel consisting of PVA/SA/Lig-Ag NPs, which exhibits low environmental toxicity, favorable mechanical properties, high hydrophilicity, and potent antimicrobial activity. The incorporation of green, low-toxicity Lig-Ag NPs can confer the hydrogel with outstanding antibacterial characteristics. Simultaneously, the composite hydrogel, comprising the polymers PVA and SA, confers the hydrogel with both stretchability and elasticity, while also enhancing its hydrophilicity and biocompatibility. The study focused on the morphology, characterization, tensile properties, rheological properties, and swelling properties of the prepared hydrogel. Additionally, we evaluated the antibacterial properties against Gram-negative bacteria and Gram-positive bacteria.

## 2. Result and Discussion

### 2.1. Hydrogel Formation

**Figure 1:**
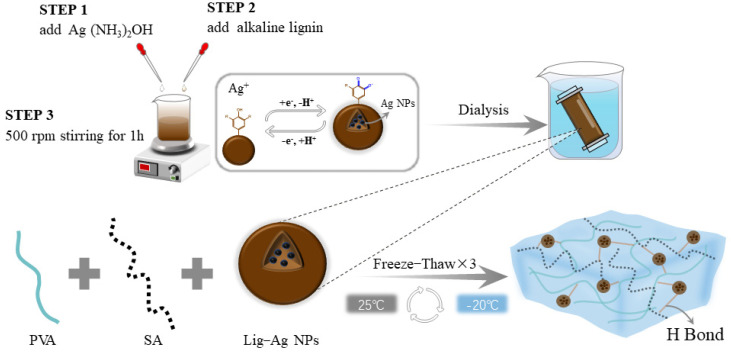
The preparation process and structural depiction of Lig-Ag NPs and PVA/SA/Lig-Ag NPs hydrogel.

The steps for environmentally friendly Lig-Ag NPs synthesis and a straightforward process for preparing composite hydrogels are depicted in Figure 1. Firstly, alkaline lignin dissolved in NaOH is slowly dripped into Ag(NH_3_)_2_OH. The interaction between phenolic hydroxyl (PhOH) and methoxyl groups (PhOCH_3_) found in lignin and Ag^+^ initiated a redox reaction throughout this process, giving rise to the formation of regular spherical nanoparticles. These nanoparticles exhibited Ag cores encapsulated within a lignin coating. Second, the mixed solution of PVA and SA was fully blended with dialyzed Lig-Ag NPs. After three freeze–thaw cycles, the PVA/SA/Lig-Ag NPs nanocomposite hydrogel was obtained. Multiple hydrogen bonds were formed between the PVA/SA/Lig-Ag NPs hydrogels through physical cross-linking, which contributed to the formation of the internal network within the gel, resulting in the construction of an antimicrobial hydrogel with favorable mechanical properties. Hereafter, the synthesized hydrogels are further discussed through a series of characterizations and performance evaluations.

### 2.2. Characterization of Hydrogels

**Figure 2:**
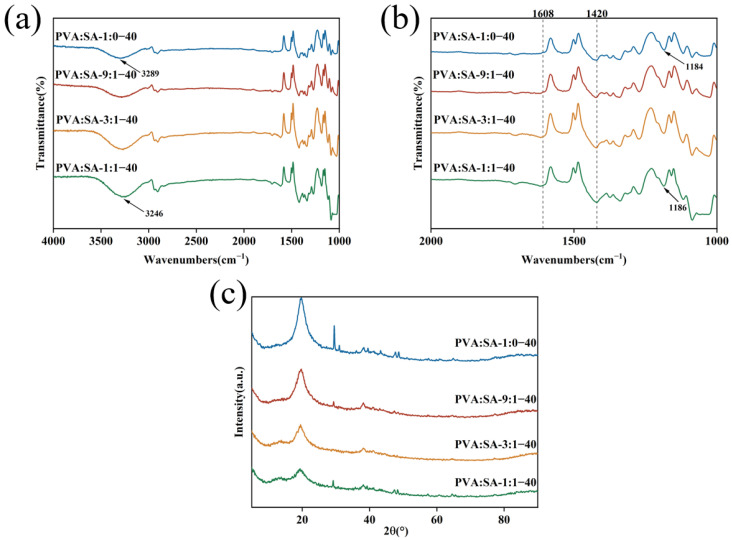
FT-IR spectra (**a**,**b**), XRD measurement (**c**) of PVA/SA/Lig-Ag NPs hydrogels.

FT-IR spectroscopy was employed to demonstrate the successful fabrication of PVA/SA/Lig-Ag NPs composite hydrogel materials and the formation of hydrogen bonds within the hydrogel. As shown in Figure 2a,b, the characteristic absorption bands of SA at 3289 cm^−1^, 1608 cm^−1^, and 1420 cm^−1^ correspond to hydroxyl (-OH) peaks, asymmetric and symmetric stretching vibration peaks of the carbonyl group (C=O) bond of the carboxylic acid group [9,31], and the increase in intensity and area of these peaks with the percentage of SA confirmed the higher content of SA in the hydrogel. The peak at 1184cm^−1^ can be attributed to the presence of PhOH on Lig-Ag NPs, further indicating the successful bonding of Lig-Ag NPs with PVA/SA compound. Meanwhile, the spectrum displayed a shift of the peak at 3289 cm^−1^ to the lower band and a shift of the peak at 1184 cm^−1^ to the higher band. The observed phenomenon suggested the formation of hydrogen bonding within the gel matrix, involving interactions between the carboxyl group of SA and the PhOH on the nanoparticles, as well as between the hydroxyl group on the PVA. The formation of hydrogen bonds can enhance the stability of the network structure within the hydrogel and contribute to the improvement of its mechanical properties [32].

Figure 2c shows the XRD spectra of different types of PVA/SA/Lig-Ag NPs hydrogels. Prominent peaks were observed at 2θ = 19.8° angles corresponding to the (101) crystal plane, which is the derivative peak of PVA, and the crystalline phase is induced by hydrogen bonding between the hydroxyl groups. Therefore, after increasing the SA proportion, there was a subsequent reduction in the intensity of the peak at 2θ = 19.8°, leading to a lower crystallinity of the hydrogel. Previous research has demonstrated the absence of diffraction peaks in pure SA [22], and as a result, no significant peaks were observed in the hydrogel after the addition of SA. On the other hand, hydrogen bonding between PVA, SA, and Lig-Ag NPs also hindered the directional alignment of the polymer molecules, which was detrimental to crystallization [33]. Meanwhile, the appearance of the characteristic peak at 2θ = 38.18° in the XRD analysis corresponded to the Lig-Ag NPs peak (Figure S1), providing evidence for the effective integration of Lig-Ag NPs into the polymer matrix.

**Figure 3:**
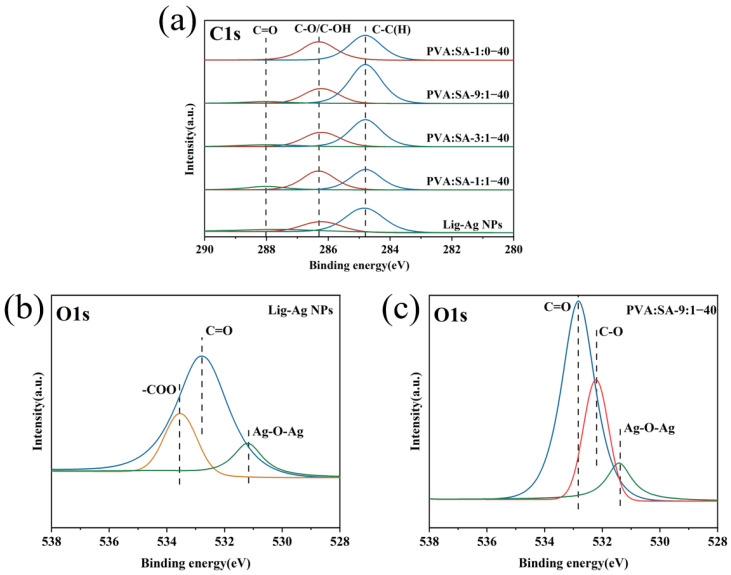
XPS spectra of C1s orbital (**a**) of PVA/SA/Lig-Ag NPs hydrogels and Lig-Ag NPs, XPS spectra of O1s orbital of Lig-Ag NPs (**b**), XPS spectra of O1s orbital of PVA:SA-9:1–40 hydrogels (**c**).

XPS analysis was used to further investigate the elemental composition and chemical state of the samples. As shown in Figure 3a, it is evident from the C1s spectra that as the SA content in the hydrogel increases, the amount of the carbonyl group (C=O) of SA at 287.7 eV also increases accordingly. The presence of C=O on Lig-Ag NPs indicated the oxidation of lignin by Ag^+^, and the C=O (532.76 eV) bond of lignin silver is observed in the O1s spectra again. Moreover, Ag–O–Ag (531.19 eV) in both the PVA:SA-9:1–40 hydrogel and hybrid nanoparticles could be attributed to hydrogen bonding interactions occurring between Ag NPs and lignin. The disappearance of the –COO (531.19 eV) peak in the O1s spectrum of the PVA:SA-9:1–40 hydrogel, in contrast to Lig-Ag NPs, might be attributed to hydrogen bonding interactions between the Lig-Ag NPs and the polymer chains. This observation is consistent with the results of the FT-IR spectral analysis.

**Figure 4:**
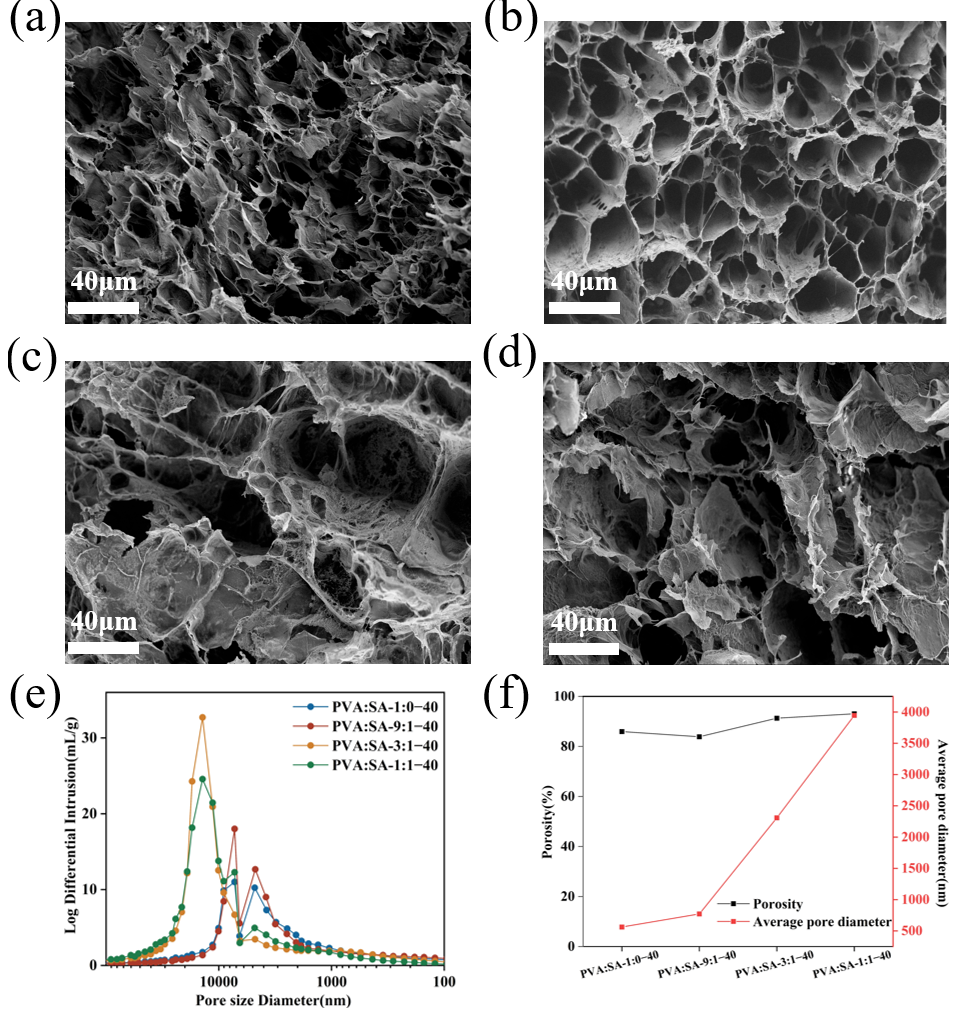
FESEM images of PVA:SA-1:0–40 (**a**), PVA:SA-9:1–40 (**b**), PVA:SA-3:1–40 (**c**), PVA:SA-1:1–40 (**d**) hydrogels, pore size distributions (**e**), porosity and average pore diameter (**f**) of PVA/SA/Lig-Ag NPs hydrogels.

The microstructures of nanomaterial composite hydrogels were analyzed by FESEM as shown in Figure 4a–d, the PVA:SA-1:0–40 and PVA:SA-9:1–40 hydrogels exhibited dense and uniform pores. However, the network structure of PVA:SA-3:1–40 and PVA:SA-1:1–40 hydrogels was changed considerably, with some macropores appearing. The adjustment of the internal porosity of the gel network was additionally verified through piezometric mercury analysis. Figure 4e,f revealed a significant enlargement in the primary pore size of the hydrogel with the increase in SA content. Additionally, the porosity of the hydrogel showed a rising trend, accompanied by a substantial increase in the average pore size from 561.97 nm to 3946.17 nm. This phenomenon can be attributed to the unique molecular structure and strong hydrophilicity of SA, which cause the relaxation of the internal mesh structure of the polymer. The presence of a dense microporous structure is expected to confer high mechanical properties [34], as will be elaborated in the subsequent sections.

**Figure 5:**
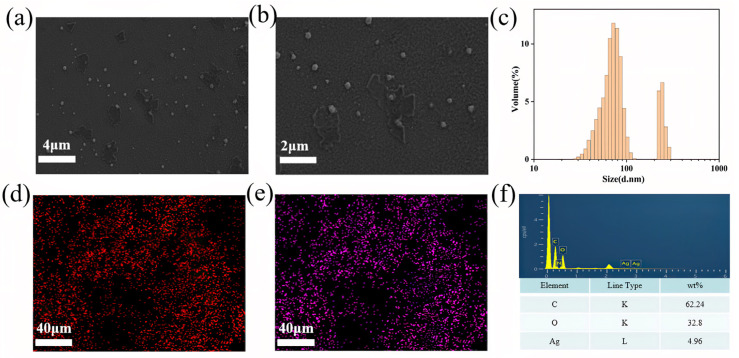
FESEM images (**a**,**b**) and DLS analysis (**c**) of Lig-Ag NPs, element mapping of carbon (**d**) and silver (**e**), EDS spectra (**f**) of PVA:SA-9:1–40 hydrogels.

The morphology of Lig-Ag NPs and their distribution on the PVA:SA-9:1–40 sample were further characterized. As shown in Figure 5a–c, the FESEM and dynamic light scattering (DLS) results revealed that the prepared Lig-Ag NPs were predominantly distributed in two primary size ranges, specifically 70 nm and 200 nm, exhibiting regular spherical structures. After the integration of Lig-Ag NPs into the hydrogel, as depicted in Figure 5d–f, carbon and silver were uniformly dispersed on the PVA:SA-9:1–40 samples, illustrating the ideal encapsulation efficiency of Lig-Ag NPs and the uniform distribution of nanoparticles.

### 2.3. Rheological Properties

**Figure 6:**
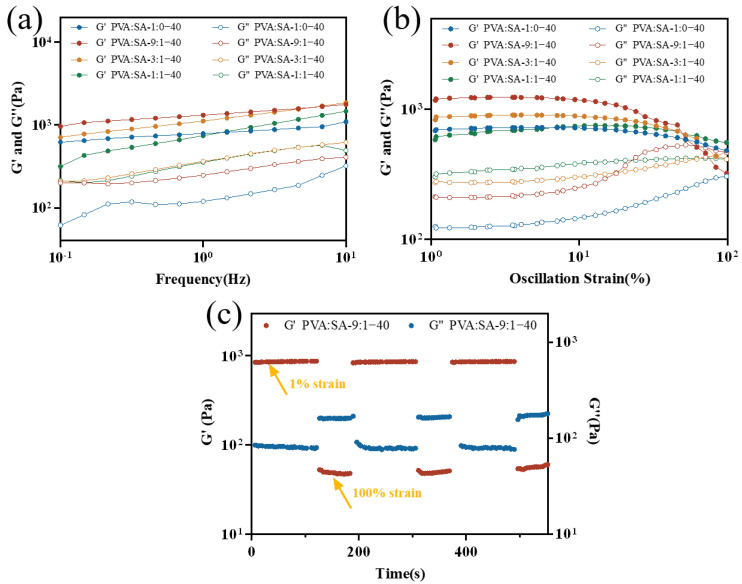
Frequency-dependent rheology measurement of the PVA/SA/Lig-Ag NPs hydrogels (**a**), strain-dependent rheology measurement of the PVA/SA/Lig-Ag NPs hydrogels (**b**), thixotropic experiment during the cyclic strain changes between 1% strain and 100% strain (**c**) of PVA:SA-9:1–40 hydrogels.

The dynamic rheological behaviors of hydrogels were revealed by both storage modulus (G′) and loss modulus (G″). G′ is related to the elastic behavior of the material, while G″ is related to the viscosity of polymers. As shown in Figure 6a, G′ with all composite samples was higher than G″ under frequency sweeps ranging from 0.1 to 10 Hz, indicating a predominance of elastic response in the rheological properties. With increasing frequency, G′ and G″ also exhibited an increasing trend attributed to dynamically reversible hydrogen bonding within the internal network [35,36]. Furthermore, the G′ value of PVA:SA-9:1–40 hydrogels was the highest among the four samples. This could be attributed to the addition of a small amount of SA, which likely resulted in an augmentation of hydrogen bonding within the hydrogel, consequently enhancing its mechanical properties. Conversely, excessive SA enlarged the pores of the gel and weakened its network structure. The strain sweep results are shown in Figure 6b, which indicated that PVA:SA-9:1–40 hydrogels also possessed the strongest mechanical properties. Alternating changes in strain (1%, 100%) were conducted to evaluate the self-healing properties of the hydrogels. G″ exceeded G′ at 100% strain, but at 1% strain, the G′ became lower than G″. When G″ was greater than G′, it indicated that collapse had occurred within the hydrogel. After the application of low stress (1%) to the hydrogels, G′ and G″ were fully recovered to their original values, demonstrating their excellent self-healing properties.

### 2.4. Mechanical Properties

**Figure 7:**
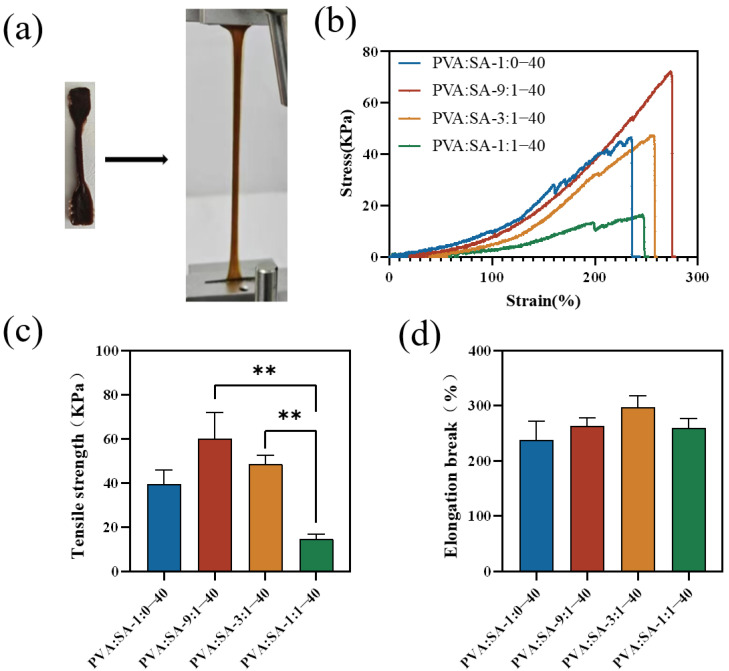
Photos of PVA/SA/Lig-Ag NPs hydrogels before and after extension (**a**), tensile stress-strain curves (**b**), tensile strength (**c**), elongation break (**d**) of PVA/SA/Lig-Ag NPs hydrogels (** *p* < 0.01).

The mechanical properties of the hydrogels were evaluated by uniaxial tensile testing, as shown in Figure 7. Among all samples, the PVA:SA-9:1–40 hydrogels showed the highest tensile strength. This result is primarily attributed to intermolecular interactions, along with the presence of small internal pore sizes within the composite hydrogel network. The findings are consistent with previous research on rheology measurement and could provide further evidence for the influence of the hydrogel internal network structure on its mechanical properties. Moreover, freeze–thaw treatment induced the formation of ordered PVA crystals via intermolecular hydrogen bonding, serving as a cross-linking mechanism that enhanced the hydrogel’s elasticity and toughness [37]. During the loading process, rearrangement and network reconstruction took place between Lig-Ag NPs and PVA chains. This phenomenon had the potential to efficiently disperse energy and delay network fracture, thereby endowing high mechanical strength and ductility to the hydrogels [38].

### 2.5. Swelling Properties

**Figure 8:**
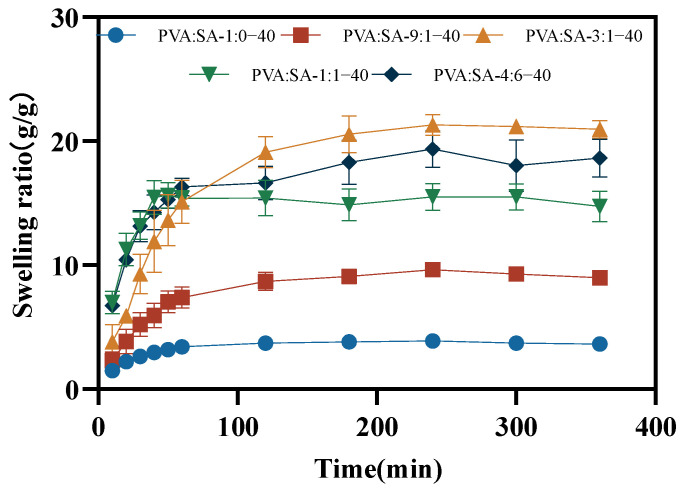
Swelling ratio of PVA/SA/Lig-Ag NP hydrogels in deionized water.

The water absorption capacity of a hydrogel was highly correlated with its practical applications. We discussed the swelling behavior of hydrogels in deionized water. As shown in Figure 8, the hydrogel rapidly absorbed water in a short period of time and gradually reached equilibrium over time, indicating the strong hydrophilicity inherent in its porous structure [9]. The accelerated swelling rate observed in the hydrogel with an escalating content of SA could be ascribed to the abundant presence of –OH and –COO· in SA. The smaller pores in the hydrogel posed a barrier to the diffusion of water molecules, resulting in a reduced equilibrium swelling rate [39]. At the same time, previous studies have shown that electrostatic repulsion caused by ionic charges triggers the swelling behavior of hydrogels [40], and the existence of Na+ in the cationic unit of SA is associated with an augmented swelling rate. Deviating from the expected results, a decrease in the swelling rate of PVA:SA-1:1–40 hydrogel occurred, and this regularity was further confirmed by the evaluation of the saturated swelling rate of PVA:SA-4:6–40 hydrogel. This phenomenon might have arisen from the disturbance in the network structure during the swelling process, resulting in the efflux of certain water molecules [41].

### 2.6. Antibacterial Properties

**Figure 9:**
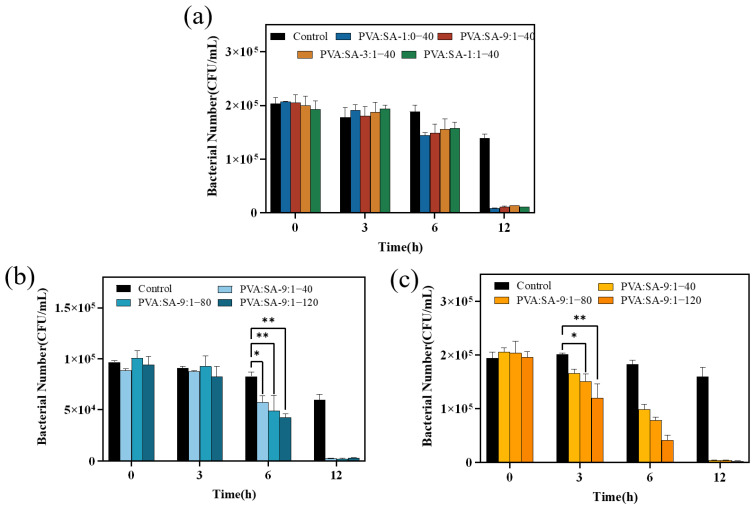
Growth inhibition on *E. coli* (**a**,**b**) and *S. aureus* (**c**) with PVA/SA/Lig-Ag NPs hydrogels (* *p* < 0.05; ** *p* < 0.01).

Initially, it was experimentally confirmed that Lig-Ag NPs have excellent antibacterial properties. 0.1 g of Lig-Ag NPs can almost completely sterilize 20 mL of *E. coli* with a bacterial count of 10^5^ CFU/mL within 6 h (Figure S2). Hereafter, the antibacterial activities of various PVA/SA/Lig-Ag NPs nanocomposite hydrogels were evaluated by incubating Gram-negative *E. coli* bacterial cells with these composite hydrogels in PBS buffer for 0, 3, 6, or 12 h. As shown in Figure 9a, the number of viable bacteria declined as the exposure time to PVA/SA/Lig-Ag NPs increased. It is noteworthy that the antimicrobial hydrogel demonstrated a 20% reduction in *E. coli* survival within 6 h and a subsequent decrease in bacterial count by an order of magnitude after 12 h of incubation (approximately 10^4^ CFU/mL). No significant differences were observed in the antibacterial activity of the four samples at different time points, suggesting that variations in the ratios of PVA and SA in the composite polymer networks did not lead to significant differences in the immobilization of Lig-Ag NPs.

The preceding discussion has established that the PVA:SA-9:1–40 hydrogel demonstrates superior mechanical properties and exhibits a water absorption capacity exceeding 5 g/g. Therefore, supplementary tests were conducted to assess the antimicrobial properties both against *E. coli* and *S. aureus*. This involved the formulation of hydrogels using a PVA:SA-9:1 ratio and the incorporation of Lig-Ag NPs with different concentrations of Ag^+^. As shown in Figure 9b,c, the number of surviving bacteria exhibited a time- and dose-dependent trend. The PVA:SA-9:1–80 and PVA:SA-9:1–120 hydrogels exhibited extremely significant antibacterial efficacy against *E. coli* within 6 h and exhibited significant antibacterial effects against *S. aureus* within 3 h. Furthermore, the PVA:SA-9:1–120 hydrogel demonstrated the best antimicrobial performance, causing the death of most bacteria within 12 h (1.63% survival for *S. aureus* and 4.47% survival for *E. coli*). The gradual release of Ag^+^ from Lig-Ag NPs, in conjunction with the quinone/semiquinone radical generation process, induced the accumulation of reactive oxygen species, ultimately leading to bacterial cell damage and death [29].

## 3. Conclusions

The present study described a straightforward method for the preparation of a PVA/SA/Lig-Ag NPs nanomaterial composite antibacterial hydrogel with excellent mechanical properties. The successful synthesis and characterization of the prepared hydrogels were demonstrated through the application of a broad spectrum of analytical techniques. Initially, a certain amount of SA was combined with PVA and Lig-Ag NPs, enhancing the mechanical strength of the hydrogel by augmenting crosslinking within the network. Simultaneously, the remarkable water absorption capacity of the hydrogel was facilitated by the hydrophilic characteristics inherent in both PVA and SA. It is noteworthy that the environmentally friendly Lig-Ag NPs endowed the hydrogel with good antimicrobial capacity, and the presence of the lignin shell on the Lig-Ag NPs contributed to low environmental toxicity. Overall, the developed hydrogels hold great promise for a variety of applications, such as healthcare and environmental protection, where both effective antibacterial properties and robust mechanical stability are crucial.

## 4. Materials and Methods

### 4.1. Materials and Chemicals

Alkaline lignin was kindly supplied by Shandong Sun Paper Industry Joint Stock Co., Ltd. (Jining, China). Silver nitrate (AgNO_3_) and polyvinyl alcohol (PVA, 98.0–99.8% hydrolyzed) were purchased from Sinopharm Chemical Reagent Co., Ltd. (Shanghai, China). Ammonium hydroxide (NH_3_·H_2_O) and sodium hydroxide (NaOH) were obtained from Damao Chemical Reagent Co., Ltd. (Tianjin, China). Sodium alginate (SA) was provided by Rhawn Chemical Technology Co., Ltd. (Shanghai, China). Luria-Bertani broth (LB) was purchased from Sangon Biotech Co., Ltd. (Shanghai, China). Phosphate buffered saline (PBS, pH = 7.2~7.4) was purchased from Solarbio Science & Technology Co., Ltd. (Beijing, China).

### 4.2. Synthesis of Lig-Ag NPs

Lig-Ag NPs were synthesized using the nanoprecipitation method in an alkaline environment with a reduction process. First, 0.25 g of basic lignin was dissolved in 5 mL of NaOH solution (5% *w*/*v*) by ultrasonic treatment to obtain solution A. Subsequently, 10 mL of AgNO_3_ solution (40, 80, and 120 mM Ag^+^) was slowly added into 5 mL (0.175 g/mL) of NH_3_·H_2_O under magnetic stirring to obtain solution B. Afterwards, solution A was slowly added to solution B under magnetic stirring for 1 h. Finally, the product was dialyzed in deionized water for 72 h and the volume was fixed to 100 mL with ionized water.

### 4.3. Preparation of PVA/SA/Lig-Ag NPs Hydrogels

PVA/SA mixed solutions with a constant total mass fraction (10 wt%) were prepared, the ratio of the mass fraction of PVA to SA was 1:0, 9:1, 3:1, and 1:1, respectively. PVA was dissolved and stirred with deionized water at 90 °C for 1 h, and then SA powder was added and dissolved at 60 °C with continuous stirring for 3 h to obtain the PVA/SA solution. Lig-Ag NPs (40 mM Ag^+^) and PVA/SA were mixed and stirred at a mass ratio of 1:1 for 1 h. The mixture was then poured into a mold and subjected to three freeze–thaw cycles between –20 °C (8 h) and 25 °C (2 h). For simplicity, these nanocomposite hydrogels were denoted as PVA:SA-x:y, with x:y representing the ratio of PVA to SA mass fraction and 40 representing the concentration of Ag^+^ in Lig-Ag NPs.

Meanwhile, in accordance with the method outlined earlier, hydrogel complexes were also prepared by thoroughly mixing Lig-Ag NPs with concentrations of 40 mM, 80 mM, and 120 mM Ag^+^ with the hydrogel mixture having a PVA to SA ratio of 9:1. The hydrogels were named PVA:SA-9:1–40, PVA:SA-9:1–80, and PVA:SA-9:1–120 based on the corresponding Ag^+^ concentrations.

### 4.4. Characterization

The chemical structure was corroborated by Fourier transform infrared spectroscopy (FT-IR), X-ray diffraction (XRD), and X-ray photoelectron spectroscopy (XPS). The prepared hydrogel samples were freeze-dried (FDU-1200, EYELA, Tokyo, Japan) before being tested for characterization. The infrared spectra of the freeze-dried hydrogels in the range of 1000–4000 cm^−1^ were measured using an FT-IR spectrometer (Nicolet iS5, Thermo Scientific Co., Ltd., Waltham, MA, USA) operating at a nominal resolution of 4 cm^−1^. A total of 16 scans were averaged to obtain each spectrum. Second-order derivative spectra were obtained using Nicolet 8.2 software (DR2, Thermo Scientific Co., Ltd., Waltham, MA, USA). XRD patterns of both the freeze-dried hydrogels and Lig-Ag NPs powders were measured using an X-ray diffractometer (D8 ADVANCE, Bruker, Karlsruhe, Germany) with diffraction angles of 5° to 90° for 2θ. The pre-treatment method for XPS samples of hydrogels and Lig-Ag NPs is identical to that of XRD. XPS analysis of the samples was detected by a photoelectron spectrometer (EscaLab Xi+, Thermo Scientific Co., Ltd., Waltham, MA, USA) using non-monochromatized Al Kα radiation (1486.6 eV). The instrument utilized a radiation source operating at 14.4 kV and 13.6 mA, while maintaining a vacuum pressure of approximately 8 × 10^−10^ Pa. The binding energy scale was corrected by referring to the C1s spectrum as being 284.80 eV.

The microscopic features of the samples were observed through field emission scanning electron microscopy (FE-SEM). The hydrogel samples were freeze-dried, then coated with platinum on the cross sections, and FE-SEM analysis (Sigma 300, Zeiss, Oberkochen, Germany) at a 5.00 kV accelerating voltage was performed. Lig-Ag NPs samples were prepared by depositing a diluted and dispersed solution onto a silicon chip at room temperature. After air-drying, the samples were coated with platinum before being subjected to FESEM analysis (Sigma 300, Zeiss, Oberkochen, Germany) at an accelerating voltage of 3 kV. The particle size was analyzed by a dynamic light scattering particle size analyzer (DLS, Malvern Nano-ZS Zeta Sizer, Malvern Instruments, Malvern, UK). Prior to analysis, the Lig-Ag NPs solution was diluted with deionized water and subjected to ultrasonic dispersion for 1 min. Elemental mapping analysis utilized energy-dispersive spectroscopy (EDS, Ultim Extreme, Oxford Instrument, Oxford, UK). The pore size and its distribution were determined by a fully automated mercury intrusion porosimeter (MIP, Autopore IV 9520, Micromerities, Waltham, MA, USA), with the samples subjected to drying and processing at 60 °C under a maximum pressure of 33,000 psi.

### 4.5. Mechanical and Rheological Measurements

The dynamic rheometer (HAAKE MARS 60, Thermo Scientific Co., Ltd., Waltham, MA, USA) was used to evaluate the dynamic rheological behavior of hydrogels. The working gap was set at 1.5 mm, and the testing temperature was 25 °C. The storage modulus (G′) and loss modulus (G″) of the hydrogels were determined at a strain rate of 1%, and a shear frequency range of 0.1–10 Hz. The strain-related modulus of the samples was determined at a shear frequency of 1 Hz, and at a strain range of 1–100%. Alternating leap strain experiments were performed at a shear frequency of 1 Hz with strains ranging from 1% to 100% as a function of time.

A mechanical testing machine (HF-9002, Li Gao, Beijing, China) was used to determine the tensile properties of hydrogels. Test samples were obtained by pouring the pre-solution of hydrogel after mixing into a dumbbell-shaped mold for freeze–thaw cycles (the experimental area was 5 mm long and 4 mm wide). Uniaxial tensile tests were conducted at a tensile rate of 30 mm/min.

### 4.6. Swelling Test

Dried hydrogels were placed in deionized water and allowed to swell for 0, 5, 10, 15, 20, 25, or 30 min, or 1, 2, 3, 4, 5, and 6 h. Subsequently, surface water present on these samples was meticulously eliminated by filter paper. The weight-to-*swelling ratio* was calculated using the following equation:

(1) $$Swelling\;ratio(g/g)=\frac{W_{s}-W_{D}}{W_{D}}$$

where *W_S_* signifies the weight of hydrogels after swelling in deionized water, and *W_D_* represents the weight of dried hydrogels.

### 4.7. Antibacterial Evaluation

*E. coli* K12-MG1655 (ATCC 700,926) was cultured in LB liquid medium for 10 h at 10^8^ CFU/mL. The medium was washed away by centrifugation and resuspension three times with PBS, after which the bacterial solution was diluted with PBS to 10^5^ CFU/mL. *Staphylococcus aureus* (*S. aureus*, ATCC 6538) was cultured in LB liquid medium for 4 h to 10^5^ CFU/mL. The medium was washed away by centrifugation and resuspension three times with PBS. 0.2 g of the hydrogel sample was placed in a 20 mL bacterial suspension containing 10^5^ CFU/mL of *E. coli*/*S. aureus*, and the system was placed in a 37 °C constant temperature incubator. 0.5 mL of the bacterial solution was taken out at different times (0, 3, 6, 12 h), diluted to a suitable magnification, and placed on a plate, and the number of colonies was obtained after incubation in LB solid medium at 37 °C for 24 h.

### 4.8. Statistical Analysis

All statistical analyses were conducted using Prism 8.3.0 software (GraphPad, La Jolla, CA, USA). Results are presented as the mean ± standard deviation (SD). One-way analysis of variance (ANOVA), coupled with the Duncan test, was performed to ascertain the significance of the difference at *p* < 0.05 and the extremely significant difference at *p* < 0.01 among the tested groups. All experiments were conducted independently, with at least triple replicates for biology and counting.

## Data Availability

The original contributions presented in the study are included in the article, further inquiries can be directed to the corresponding authors.

## Supplementary Materials

The following supporting information can be downloaded at: https://www.mdpi.com/article/10.3390/gels10040240/s1, Figure S1: XRD measurement of Lig-Ag NPs; Figure S2: Growth inhibition on *E. coli* with Lig-Ag NPs; Figure S3: Photos of the live bacteria colony on solid LB agar plates exposure to PVA:SA-9:1-80 hydrogel for different durations; Table S1: Comparison of antimicrobial properties of PVA/SA/Lig-Ag NPs hydrogels with other hydrogels containing Ag NPs (Refs. [42,43,44,45,46,47,48] were cited in Supplementary Materials).
