# Supplementary material for: A Lignin Silver Nanoparticles/Polyvinyl Alcohol/Sodium Alginate Hybrid Hydrogel with Potent Mechanical Properties and Antibacterial Activity
Source: Gels. 2024 Apr 1;10(4):240. doi: 10.3390/gels10040240 (PMC11049037; doi:10.3390/gels10040240)
Supplement: Supplementary file 1 [file gels-10-00240-s001.zip › gels-2915086-supplementary.pdf]

Supporting Information

A lignin silver nanoparticles/polyvinyl alcohol/sodium alginate hybrid hydrogel with potent mechanical properties and antibacterial activity

Jie Yu[1,2†], Fangli Ran[2,3†], Chenyu Li[2*], Zhenxin Hao[2,3], Haodong He[2,3], Lin Dai[3], Jingfeng Wang[2*], Wenjuan Yang[1*]

[1]Key Laboratory of Subsurface Hydrology and Ecological Effect in Arid Region of the Ministry of Education, School of Water and Environment, Chang'an University, Xi'an 710064, China

[2]Department of Environment and Health, Institute of Environmental and Operational Medicine, Tianjin 300050, China

[3]Tianjin Key Laboratory of Pulp and Paper, Tianjin University of Science and Technology, Tianjin 300457, China

*Corresponding author

†These authors have contributed equally to this work.

Tel: +86 22 84655052

Fax: +86 22 23328809

E-mail: ywj_xa@chd.edu.cn (W.Y.),

nk_lcy710430@hotmail.com (C.L.)

Table of Contents

1. Experimental Procedures

Measurement of Growth inhibition on *E. coli* with Lig-Ag NPs.

2. Supplementary Figures

Fig. S1. XRD measurement of Lig-Ag NPs.

Fig. S2. Growth inhibition on *E. coli* with Lig-Ag NPs.

Fig. S3. Photos of the live bacteria colony on solid LB agar plates exposure to PVA:SA-9:1-80 hydrogel for different durations.

3. Supplementary Figures

Table S1. Comparison of antimicrobial properties of PVA/SA/Lig-Ag NPs hydrogels with other hydrogels containing Ag NPs.

1. Experimental Procedures

Measurement of Growth inhibition on *E. coli* with Lig-Ag NPs. The sample was Lig-Ag NPs, with Ag^{+} concentration of 40 mM during the preparation of silver from lignin. In the antibacterial evaluation, 0.1 g of lignin silver suspension was taken and mixed with 20 mL of diluted bacterial suspension (*E. coli* bacterial load of 10^{5} CFU/mL), and the remaining bacterial count in the suspension was assessed at 0, 3, 6, and 12 hours. The pre-treatment and bacterial enumeration methods for bacterial suspensions were conducted in accordance with the hydrogel antibacterial assay protocol.

2. Supplementary Figures

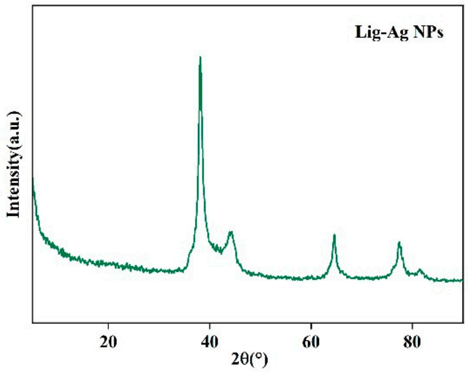

Fig. S1. XRD measurement of Lig-Ag NPs.

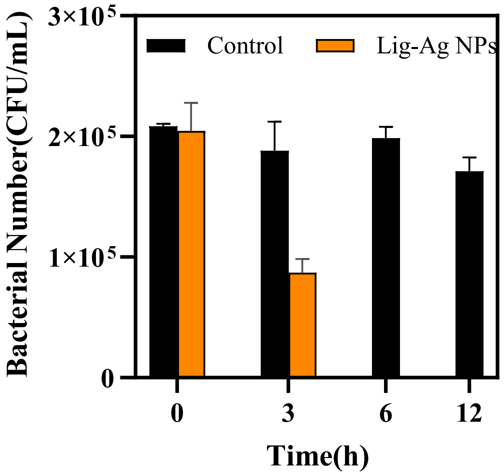

Fig. S2. Growth inhibition on *E. coli* with Lig-Ag NPs.

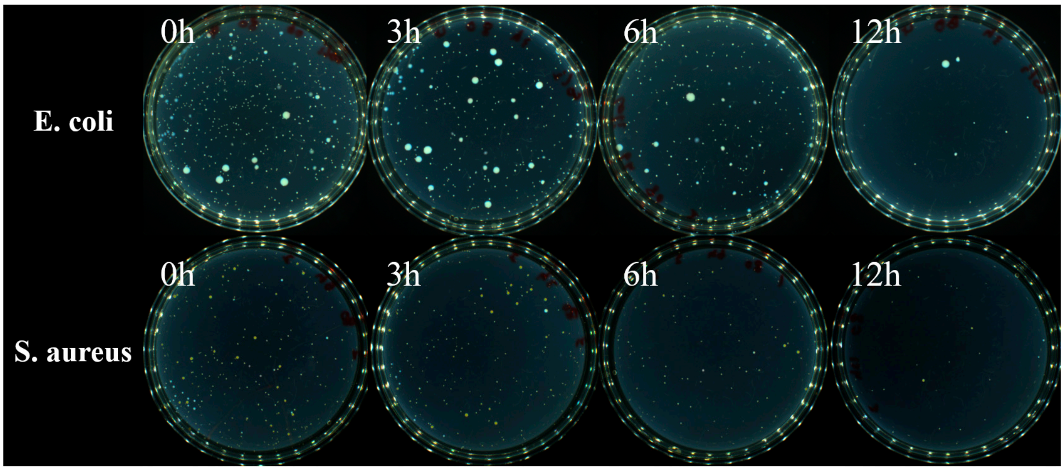

Fig. S3. Photos of the live bacteria colony on solid LB agar plates exposure to PVA:SA-9:1-80 hydrogel for different durations.

3. Supplementary Figures

Table S1. Comparison PVA/SA/Lig-Ag NPs hydrogels with other hydrogels.

No.	Materials	the antimicrobial substances	Test microorganism	The antibacterial ratio of the hydrogel	Ref.
1	ATC/SA/PVA hydrogel	Ag NPs	*Escherichia coli*	97.1%	[42]
			Staphylococcus aureus	95.9%	[42]
2	SS/PNIPAAm/Ag NPs IPN hydrogel	Ag NPs	*Escherichia coli*	95%	[43]
			Staphylococcus aureus	97%	[43]
3	Gel/PP-TA-Ag hydrogel	Ag NPs, TA	*Escherichia coli*	70.56%	[44]
			Staphylococcus aureus	75.52%	[44]
4	Ag NPs@MIL-100(Fe)/ GG hybrid hydrogel	Ag NPs, MIL100(Fe)	*Escherichia coli*	99.6%	[45]
5	SS/NIPAAm-Ag_3 hydrogel	Ag NPs	*Escherichia coli*	99.8%	[46]
			Staphylococcus aureus	93.4%	[46]
6	C_3N_4-PDA-Ag@CS composite film	C_3N_4, Ag NPs	*Pseudomonas aeruginosa*	＞80%	[47]
			Staphylococcus aureus	＞80%	[47]
7	Ch-3/Ag NPs hydrogel	Ch, Ag NPs	*Escherichia coli*	＞90%	[48]
			Staphylococcus aureus	＞90%	[48]
This work	PVA/SA/Lig-Ag NPs hydrogel	Lig-Ag NPs	*Escherichia coli*	95.53%	
			Staphylococcus aureus	98.37%	
